# Supplementary material for: Microbial community succession mediated by planting patterns in the Loess Plateau, China: Implications for ecological restoration
Source: PLoS One. 2025 May 27;20(5):e0324786. doi: 10.1371/journal.pone.0324786 (PMC12112373; doi:10.1371/journal.pone.0324786)
Supplement: Attachment — Submitted filename: PLOSone review (Autosaved).docx [file pone.0324786.s001.docx]

**Reviewer Report - PONE-D-24-15257**

**Title:** Microbial community succession mediated by planting patterns in the loess beam-shaped hilly region of China

**Reviewer**: Bright Fafali Dogbey

**General Overview:**

This manuscript addresses an important ecological issue by examining the effects of planting patterns on microbial community succession and soil function in the degraded Loess Plateau region of China. The study employs next-generation sequencing (NGS) and soil metagenomics to provide valuable insights into microbial diversity and function during restoration processes. The findings are highly relevant to land management and ecological restoration efforts, particularly in degraded ecosystems.

The manuscript is well-structured and covers significant data, but some areas require further clarification and elaboration. Improvements in presentation, explanation of ecological relevance, and methodological details will enhance the clarity and impact of the paper. Below is a detailed breakdown of strengths and areas for improvement in each section.

**Specific Comments:**

1. **Title**

**Strengths**:

**Descriptive and Specific**: The title is clear and provides a strong sense of what the paper is about. It describes the core focus of the study, which is microbial community succession, and specifies the geographic region (Loess beam-shaped hilly region in China). This helps readers understand the subject matter and location immediately.

**Focus on Key Terms**: The title includes key terms such as "microbial community succession" and "planting patterns," which accurately reflect the study's focus. This will help the manuscript be discoverable by readers interested in these topics.

**Suggestions for Improvement**

- **Clarification of "Loess beam-shaped hilly region"**: The phrase "loess beam-shaped hilly region" might be confusing or overly technical for some readers. Consider simplifying this or replacing it with a more widely understood term, such as "Loess Plateau" (if applicable), or include a more descriptive term like “hilly areas of the Loess Plateau.” A clearer alternative could be: "Microbial community succession mediated by planting patterns in the Loess Plateau, China."
- **Emphasize Novelty or Key Findings**: You might suggest adding a phrase that highlights the unique contribution of the paper or key findings. For example, something like "Microbial community succession and its role in ecological restoration..." could emphasize the ecological significance of the study.

**Suggested Title Revision**: “Microbial Community Succession Mediated by Planting Patterns in the Loess Plateau, China: Implications for Ecological Restoration”

### 2. Abstract Review

**Strengths:**

- **Clear Objective:** The abstract clearly states that the study investigates microbial community succession across different planting areas in the Loess Plateau region using soil metagenomics.
- **Key Findings Included:** The results section of the abstract does summarize the major findings, such as the distribution of carbon (C) and nitrogen (N) being highest in mixed planting areas and bacteria showing more variability than fungi.
- **Structured Presentation**: The abstract follows a logical structure, introducing the problem, methodology, results, and conclusions in a concise manner.

**Suggestions for Improvement:**

**Provide Specific Data Points**: The abstract would be stronger with the inclusion of specific data points or percentages from the study’s findings. For example, instead of saying "bacteria showed higher succession variability," it could specify by how much, and give concrete differences between mono- and mixed-planting areas.

**Clarify the Methodology:** While the use of metagenomics is mentioned, it's important to briefly explain what it entails for readers unfamiliar with the term. A simple phrase like “using high-throughput sequencing techniques” would help make this clear.

**Expand on Ecological Implications**: The final sentence of the abstract discusses the “broad strategic significance” of mixed cultivation for weakened ecologies. This could be expanded slightly to provide more context on how the findings can be applied in real-world restoration efforts, or mention the broader implications in land management or ecosystem health.

3. **Introduction Review**

**Strengths:**

- **Good Contextualization of the Study**: The introduction provides an excellent background on the issue of ecological degradation in the Loess Plateau and how microbial communities play a role in soil health and restoration. It effectively positions the study within the larger context of ecological research and the need for vegetation restoration.
- **Relevant Literature Cited**: The authors reference several important studies related to the Grain-for-Green Program (GFGP) and past afforestation efforts in China. This helps build a solid foundation for the study.

**Suggestions for Improvement:**

- **Clear Statement of Research Gap**: While the introduction outlines the general ecological issues in the Loess Plateau, the specific research gap regarding microbial community succession is somewhat vague. It would help to more explicitly state what is currently unknown about microbial succession in relation to different planting patterns and why this study is needed. A possible statement could be: “Although previous research has explored vegetation restoration on degraded soils, the specific mechanisms through which different planting patterns affect microbial community succession and soil function remain unclear.”
- **Hypothesis or Research Questions**: The introduction would benefit from a clear articulation of the research questions or hypotheses. This would help guide the reader through the study. For instance, “This study hypothesizes that mixed planting patterns will promote greater microbial diversity and soil function restoration than mono-planting patterns.”
- **Streamline Information on Past Programs**: The background on deforestation, overgrazing, and previous restoration programs is valuable but can be condensed. The introduction could focus more on the direct impact of these activities on microbial communities rather than spending too much time discussing broader deforestation issues.

The paragraph beginning with "Various microorganisms with different functional characteristics in the soil..." (lines 40-43) has a complex structure. Simplifying this section and focusing on the functional importance of microbial communities in relation to the study would make it more accessible.

There are minor grammar issues throughout, such as the overuse of commas in some sentences (e.g., lines 51-54). These could be streamlined for better readability.

**Materials and Methods Review**

**Strengths:**

- **Comprehensive Methodology**: The methodology section is detailed and includes various soil properties (pH, enzyme activities, C, N, etc.) as well as microbial sequencing data, which is essential for understanding the scope of the study.
- **Use of Modern Techniques**: The use of high-throughput sequencing (Illumina MiSeq) and statistical analyses (ANOSIM, PCoA) demonstrates that the study employs up-to-date and powerful tools for microbial community analysis.

**Suggestions for Improvement:**

**Clarify Sampling Procedures**: The methodology could be more explicit about the exact number of replicates per planting type and sampling location. Were the samples collected from the same depth consistently across all sites? This detail will improve the transparency and reproducibility of the study.

**More Information on Sequencing Quality Control**: The manuscript briefly mentions sequencing techniques but doesn't delve into the quality control processes used to ensure reliable data. Adding a section on how low-quality sequences were filtered out and how sequencing depth was standardized would enhance confidence in the findings.

**Justification of Statistical Methods**: While the methods mention using ANOVA and MetagenomeSeq, it would be helpful to explain why these particular methods were chosen and whether alternative statistical tests were considered. For example, why was MetagenomeSeq selected over other differential abundance analysis tools, and how was significance determined?

The DNA extraction method (lines 134-138) could provide more information on why PowerSoil® DNA Kit was selected and how it compares with other kits.

The qRT-PCR process is well explained, though it might help to specify why 35 cycles were chosen for the PCR process (lines 141-145).

**Study Area and Soil Sampling (Lines 91–105):**

"This research was conducted in the Zhongzhuang Village Forest Farm in Baiyang Township, Pengyang County, Guyuan City, Ningxia Hui Autonomous Region, China...". The study area is well-described, with specific geographic coordinates provided. This adds credibility and allows for reproducibility. The selection of eight cultivation areas based on geomorphological conditions is appropriate and ensures consistency across sampling sites.

- **However,** the description of the landform type “Liangmao hilly land in the belly of the Loess Plateau” could be clearer. Terms like "belly" may not be intuitive. Consider revising this phrase to something more precise, such as "the central region of the Loess Plateau."
- The statement “The temporal change was usually lower than the spatial change in a few cases…” (line 101) is unclear. It would help to explain why temporal changes were less significant and provide a brief rationale for focusing on spatial variations.
- It would be useful to explain why a depth of 0–0.20 m was chosen for soil sampling, especially in the context of microbial studies. While it is a common practice to sample surface soil, a brief justification would add depth to the methods.

**Soil Sampling and Sample Preparation (Lines 106–120):**

"Surface soil samples (0–0.20 m depth) were collected from plant-growing areas at the eight selected locations..."

The method of selecting sample plots (20 m × 20 m) and setting three small plots within them is clear and appropriate for obtaining representative samples.

The use of a metal soil core sampler ensures the integrity of the samples, which is important for microbial and physicochemical analysis.

The term "typical plants were selected" (line 111) is vague. It would help to specify what criteria were used to select these plants (e.g., dominance in the area, species used in the restoration project).

The rationale for using three replicate plots could be explained more explicitly, even though replication is standard practice in ecological studies. Explaining how this contributes to statistical reliability would strengthen the methods.

**Determination of Soil Physicochemical and Enzymatic Properties (Lines 121–132):**

"The soil moisture content was determined by the drying method, and the soil bulk density was determined by sampling and weighing with a ring knife..."

- **Strengths**:
  - The section is well-organized, and standard methods for determining soil properties such as moisture content, bulk density, pH, and organic carbon are employed.
  - The use of specific methods like the dichromate oxidation for SOC and Kjeldahl digestion for TN demonstrates adherence to well-established procedures.
- **Areas for Improvement**:
  - The enzymatic activity assays (sucrase, cellulase, urease, etc.) are well described, but it would be helpful to briefly mention why these particular enzymes were chosen. Are they linked to specific ecological functions or restoration processes?
  - Consider providing a reference or rationale for using potassium permanganate for catalase activity and iodometric titration for peroxidase and polyphenol oxidase. Even though these are standard methods, it would help to briefly state their relevance to microbial community functions.

**Physicochemical Analysis - Additional Recommendations**

In the section where the authors describe the physicochemical analysis of the soil, they should provide:

1. **Citations to Established Protocols**: Each method used for measuring soil properties, such as pH, organic carbon, total nitrogen, and enzymatic activities, should be cited with references to standard or well-accepted protocols. For instance:
   - **Soil Organic Carbon (SOC)** and **Total Nitrogen (TN)**: Methods like the **Walkley-Black method** for SOC or the **Kjeldahl method** for TN should be cited with appropriate references. This ensures the readers are aware of the methodologies that follow scientific standards.
   - **Enzyme Activity Assays**: For enzyme activities such as sucrase and phosphatase, the protocols followed (e.g., 3,5-dinitrosalicylic acid for sucrase activity or the colorimetric method for phosphatase) should be clearly referenced to specific scientific papers or protocol handbooks.
2. **Details of Equipment Used**: For each measurement, the specific equipment and its manufacturer should be mentioned to enhance reproducibility. For example:
   - pH measurements should include details such as the brand and model of the pH meter used.
   - Spectrophotometric assays (e.g., for phosphatase activity) should mention the spectrophotometer used, including its model and manufacturer.

- If standard laboratory equipment (e.g., **Kjeldahl digestion apparatus** for nitrogen measurement or **muffle furnace** for organic matter analysis) was used, these should also be specified.

**Soil DNA Extraction, Amplification, and Illumina MiSeq High-Throughput Sequencing (Lines 133–157):**

"Herein, the method described by Fierer et al. was used to quantify the relative abundance of bacterial and fungal rRNA gene copies..."

- **Strengths**:
  - The use of established protocols such as those by Fierer et al. for DNA extraction lends credibility to the study. The PowerSoil® DNA kit is appropriate for this type of study, as it is widely used in soil microbiome research.
  - The detailed explanation of PCR reaction conditions and the use of MiSeq high-throughput sequencing ensures that the experimental design can be reproduced.
- **Areas for Improvement**:
  - The rationale for choosing 16S rRNA for bacteria and ITS for fungi should be briefly explained. While these are standard markers, it would help to reinforce why they are appropriate for identifying microbial communities in this study.
- **Strengths**:
  - The use of standard statistical tests like ANOVA and post-hoc Duncan multiple range tests is appropriate. Welch’s t-test is also well justified for comparing differences between two sample sets.
  - The inclusion of PCA and PCoA analyses is valuable for assessing functional diversity and community composition.
- **Areas for Improvement**:
  - The choice of statistical tests should be more clearly justified. For example, why was Welch’s t-test chosen over a standard t-test? While the explanation that it accounts for unequal variances is valid, adding a brief explanation of its relevance to the dataset would improve clarity.
  - It would be beneficial to briefly explain why functional annotations were performed using KEGG, eggNOG, Pfam, and other databases. Highlighting the relevance of these specific databases to microbial metagenomics would make this section more informative.
  - The section on metagenomics assembly and annotation (lines 165–172) is a bit technical and could be simplified for clarity. For instance, phrases like “filtering contig sequences shorter than 300bp” could benefit from a brief explanation for non-specialist readers.

**Illumina MiSeq High-Throughput Sequencing (Lines 152–157):**

- **Strengths**:
  - The description of sequencing and clustering into operational taxonomic units (OTUs) at 97% similarity is appropriate for microbial community analysis.
- **Areas for Improvement**:
  - Mentioning how the authors addressed potential sequencing errors or biases would be helpful. For example, were any quality control steps (e.g., chimera checking, sequence trimming) employed?

**Functional Gene Annotation (Lines 167–174):**

- **Strengths**:
  - The functional annotations using KEGG and eggNOG databases are well explained. These databases provide valuable insights into microbial metabolic pathways, which are relevant to the study's focus on restoration.
- **Areas for Improvement**:
  - Briefly explain why functional gene annotations are essential for this study. For example, linking these annotations to the study's broader goals (i.e., understanding microbial functions related to ecological restoration) would clarify their significance.

**Recommendations for Improvement:**

1. **Clarity and Simplification**: Simplify technical jargon where possible, especially when referring to advanced metagenomics procedures. This would make the methods section more accessible to a broader audience.
2. **Rationale for Method Choices**: Provide more detailed explanations of why certain methods were chosen. This includes the selection of specific enzymes for soil analysis, the use of 35 PCR cycles, and the selection of statistical tests.
3. **Quality Control**: Add more details on how contamination was controlled during DNA extraction and PCR processes, as well as how sequencing errors were minimized.
4. **Reorganization**: Consider reorganizing some sections for better flow. For example, the DNA extraction and sequencing sections could be combined for a more cohesive explanation of the microbial analysis.

**4. Results Review**

**Strengths:**

- **Comprehensive Data**: The results section presents a wide array of data, covering soil properties (physical, chemical, biological), enzyme activities, and microbial community composition. This thoroughness allows for a detailed comparison across planting types.
- **Use of Visuals**: Figures, such as the β-diversity analysis and enzyme activity heat maps, are valuable for illustrating complex trends. The authors have clearly put effort into presenting their data visually, which aids in understanding the differences between planting patterns.
- **Functional Analysis**: The inclusion of functional gene analysis, such as KEGG pathways and GO annotations, adds depth to the study by linking microbial community structure with potential ecological functions, such as carbohydrate and amino acid metabolism.

**Suggestions for Improvement:**

- **Clarity in Data Presentation**: While the data presented are thorough, the narrative accompanying the tables and figures is not always clear. For example, the authors could more explicitly explain what the enzyme activity data mean in the context of soil health and microbial function. The data show changes, but the ecological relevance (e.g., why higher sucrase or phosphatase activity matters for soil restoration) should be spelled out more clearly.
- **Statistical Significance**: The results should emphasize statistically significant findings more clearly. While the authors mention differences between planting types, it’s important to highlight which specific comparisons (e.g., between mono- and mixed-planting areas) showed significant differences in microbial abundance or enzyme activity. This can be achieved by including P-values or confidence intervals directly in the text or in figure legends.
- **Explain Trends in Beta-Diversity**: The β-diversity analysis, using PCoA and NMDS, is presented in figures but is not thoroughly explained. The results should describe what these analyses indicate about the microbial community structure. For instance, how much of the variation in microbial community structure is explained by planting type? Are there clear clusters that correspond to specific planting patterns?
- **Functional Gene Analysis**: The KEGG and eggNOG pathway analyses show interesting results, but the ecological relevance of these pathways needs more explanation. How does the shift in carbohydrate and amino acid metabolism contribute to the restoration of degraded soils? What role do these functions play in soil fertility or plant growth?

**Results (Lines 176–295):**

**General Comments:**

The **Results** section is thorough and presents a wide range of findings, including soil physicochemical properties, microbial composition, functional gene diversity, and microbial community diversity across different planting patterns. The data are well-organized, and the use of tables and figures is appropriate. However, there are areas where the results could be more clearly tied to the research question, and some results lack sufficient explanation or interpretation. Additionally, the figures and tables should be more directly referenced and explained within the text.

**Section-by-Section Review:**

**Soil Physicochemical and Biological Enzymatic Characteristics (Lines 176–209):**

"Chemical, physical, and biological characteristics are always selected as important soil quality indicators..."

- **Strengths**:
  - This section provides a detailed comparison of soil chemical properties under different planting patterns. The focus on key properties like total organic carbon (TOC), total carbon (TC), nitrogen species, and enzyme activities is highly relevant to the study's aims.
  - The inclusion of enzyme activities such as phosphatase and sucrase adds depth to the assessment of biological function in soils.
- **Areas for Improvement**:
  - The section could benefit from clearer connections between the changes in physicochemical properties and the microbial community changes observed. For example, how do increased TC and TOC values in multi-plant systems specifically promote microbial community diversity or functional activity?
  - The results for enzyme activities (lines 190–195) are mentioned briefly, but the ecological significance of these changes is not fully explained. Why is the decrease in sucrase and BG activity in some areas important? How do these changes affect soil microbial functions or ecosystem health?
  - The description of NO_3_–N and NH_4_^+^–N fluctuations (lines 197–198) could be more detailed. A discussion of why these nitrogen forms vary so much across different planting types would enhance the reader's understanding of the underlying mechanisms.

**Soil Microbiological Composition Under Different Cropping Patterns (Lines 211–232):**

"Classification and identification of sequencing results showed that the main composition of microorganisms was bacteria, accounting for 83.2–83.9% of all microbial groups..."

- **Strengths**:
  - The presentation of microbial composition data is clear, with a good breakdown of the dominant taxa found in different planting types. Acidobacteria and other key taxa are appropriately highlighted.
  - The use of percentages to describe the relative abundance of bacterial and fungal groups provides a straightforward way to compare the microbial communities across the different planting patterns.
- **Areas for Improvement**:
  - The results show that Acidobacteria are dominant, but the ecological importance of this dominance is not fully explained. A brief discussion of how Acidobacteria contribute to soil processes (e.g., nutrient cycling, pH regulation) would add value here.
  - The sentence "Changes in soil ecological restoration from derelict to artificial grasslands and woodlands can disrupt and affect the soil environment and communities significantly" (lines 220–221) introduces an important point but lacks sufficient detail. How do these changes manifest in the microbial community data presented? Were there specific shifts in community composition that reflect these disruptions?
  - The figures (e.g., Table 3 and Figure 3) should be more clearly referenced and explained. It would help to provide more explicit references to which part of the figure corresponds to specific findings discussed in the text.

**Functional Changes of Microbial Communities (Lines 236–272):**

"Functional annotations were performed on the acquired biological information..."

- **Strengths**:
  - The focus on functional annotations and metabolic pathways is a strong point, particularly the identification of carbohydrate metabolism, amino acid metabolism, and energy production as key microbial functions.
  - The use of databases like KEGG, eggNOG, and CAZy is appropriate, and the analysis of functional gene diversity is highly relevant to understanding the ecological roles of the microbial communities.
- **Areas for Improvement**:
  - The results mention important functional genes but lack in-depth interpretation. For example, why are genes related to carbohydrate and energy metabolism particularly important for soil restoration? A more detailed explanation of how these functions contribute to ecosystem recovery would enhance this section.
  - The correlation between the abundance of certain functional genes and changes in microbial composition should be explored further. It would help to discuss specific examples of how shifts in microbial community structure (e.g., increases in specific taxa) are linked to changes in functional gene abundance.
  - The results from Figure 4 are briefly mentioned, but the significance of these figures is not fully explained. Consider discussing the most important takeaways from the figure, such as which functional pathways are most upregulated in mixed planting systems.

**Diversity Changes of Microbial Communities (Lines 273–295):**

"The β diversity analysis of species composition under different planting types was carried out..."

- **Strengths**:
  - The use of β-diversity analysis (PCoA, NMDS, UPGMA) is well-suited to understanding the impact of planting patterns on microbial community composition. The detailed description of these analyses provides valuable insights into community shifts.
  - The results indicate that mixed planting patterns enhance microbial community stability, which is a key finding for restoration ecology.
- **Areas for Improvement**:
  - While the β-diversity results are presented well, more interpretation is needed to explain why mixed planting systems promote greater microbial diversity. For example, how do mixed plant species influence the rhizosphere environment, and why does this lead to more stable microbial communities?
  - The importance of specific microbial taxa in these diversity changes is mentioned but not deeply explored. For example, why do certain bacteria like Acidobacteria show such strong responses to planting type? Providing a more detailed ecological rationale would strengthen the analysis.
  - The correlation between microbial diversity and soil health could be made more explicit. How do changes in microbial diversity translate into changes in ecosystem functioning or resilience?

**Discussion (Lines 296–378) Review:**

**General Comments:**

The **Discussion** section does a good job of summarizing the key findings and linking them to broader ecological processes. However, the discussion could be more cohesive, with clearer connections between the study’s results and the existing literature. Some parts of the discussion are too brief and do not fully explore the implications of the findings. The ecological and practical relevance of the study could also be emphasized more strongly.

**Section-by-Section Review:**

**Microbial Community Composition and Function (Lines 296–319):**

- **Strengths**:
  - The discussion on how mixed planting patterns influence microbial composition and function is well-articulated. The link between vegetation diversity and soil microbial stability is a key takeaway from the study.
  - The statement that "multi-plant planting patterns were more conducive to soil fertility recovery" (lines 308–309) is supported by the results and is a critical point for restoration ecology.
- **Areas for Improvement**:
  - The discussion mentions that microbial community changes are linked to soil enzyme activities and nutrient content, but it lacks specific examples or references to the data presented earlier. It would help to revisit the enzyme activity results and link them to the changes in microbial communities more explicitly.
  - The ecological mechanisms driving the changes in microbial function (e.g., why carbohydrate metabolism is important) could be explained in more depth. For example, how do these functional shifts directly influence soil recovery processes?

**Implications for Soil Restoration (Lines 320–342):**

- **Strengths**:
  - The practical implications of mixed planting for soil restoration are discussed, which is important for translating the findings into actionable strategies for ecological management.
  - The link between soil microbial function and ecosystem restoration is well-established.
- **Areas for Improvement**:
  - This section could be expanded to explore the broader ecological consequences of microbial succession in more detail. For example, how do the microbial functions identified (e.g., carbohydrate metabolism) contribute to long-term soil health and plant productivity?
  - The discussion briefly touches on the importance of mixed cropping systems, but the mechanisms by which these systems promote microbial diversity and stability could be further elaborated.

**Study Limitations and Future Directions (Lines 343–378):**

- **Strengths**:
  - The acknowledgment of study limitations (e.g., temporal variability, small sample size) adds transparency and shows a critical perspective on the research findings.
- **Areas for Improvement**:
  - This section could benefit from more specific suggestions for future research. For instance, what specific microbial processes or plant-soil feedback mechanisms should future studies focus on?
  - The authors mention the importance of understanding plant-microbe interactions, but this point could be expanded. What specific aspects of these interactions are currently underexplored, and how might they be studied in the future?

**Recommendations for Improvement:**

1. **Link Results to Research Questions**: Make stronger connections between the findings and the research questions. Explicitly state how each result contributes to understanding microbial succession and soil restoration.
2. **Interpretation of Functional Data**: Provide deeper interpretations of the functional gene data and enzyme activities. Explain how these findings relate to broader ecological processes like nutrient cycling and soil health.
3. **Expand Discussion of Ecological Implications**: Explore the ecological and practical significance of the results in more detail, particularly regarding how mixed planting systems can be used in restoration efforts.
4. **Clarify the Role of Microbial Taxa**: Discuss in more depth the role of specific microbial taxa (e.g., Acidobacteria) in soil restoration and why they respond differently to various planting patterns.
5. **Broaden Discussion on Future Research**: Suggest more specific avenues for future research, particularly focusing on plant-microbe interactions and long-term soil health outcomes.

**Conclusion Review**

**General Comments:**

The **Conclusion** section provides a succinct summary of the findings, highlighting the significance of mixed planting systems in improving soil microbial diversity and stability. However, it could benefit from stronger linkages to the broader ecological implications and practical applications of the findings. Additionally, the conclusion should address future research directions and acknowledge potential limitations of the study, which would provide a more holistic closure to the paper.

**Section-by-Section Review:**

**Summary of Key Findings (Lines 362–378):**

"In this study, the effects of different restorative plantations on the soil microbial composition and structure were investigated..."

- **Strengths**:
  - The conclusion correctly emphasizes the major finding that mixed planting systems result in higher microbial diversity and functional gene abundance compared to monoculture systems. This is a strong and important outcome of the study.
  - The observation that multi-plant systems lead to greater internal stability of the soil environment is well supported by the data, making it a valid point to highlight.
- **Areas for Improvement**:
  - While the conclusion summarizes the findings, it lacks depth in explaining the broader ecological and practical relevance. For instance, how do these findings influence strategies for soil restoration, reforestation, or agricultural land management? More emphasis should be placed on the real-world implications of the results.
  - The mention that microbial abundance is 32.1% higher in mixed systems is valuable, but it would be even more impactful to explain what this increased abundance means for soil health, nutrient cycling, or ecosystem resilience.
  - The conclusion could restate the significance of microbial functions identified in the study, such as carbohydrate metabolism and energy production, and how they contribute to long-term soil recovery.

**Ecological and Practical Implications (Lines 375–378):**

"By improving the management and control of soil cultivation patterns, mixed forest areas have great potential for forming multiple dominant species, which is conducive to maintaining the stability of fragile ecological environments."

- **Strengths**:
  - The potential for mixed forest areas to promote ecological stability is highlighted well, making a case for their broader application in land restoration projects.
  - The statement that the study’s findings are “conducive to maintaining the stability of fragile ecological environments” is appropriate and demonstrates the study’s importance in the context of environmental restoration.
- **Areas for Improvement**:
  - This section could benefit from more detailed examples or case studies. For instance, how can the findings be applied to other ecologically degraded regions beyond the loess plateau? Providing a broader geographic or environmental context would strengthen the applicability of the research.
  - The conclusion could elaborate more on the specific management practices that should be implemented based on the study’s findings. What should land managers or policy makers take away from this research, and how should they modify existing strategies?

**Future Research Directions:**

- **Strengths**:
  - While the conclusion does not explicitly mention future research directions, it hints at the need for further exploration of soil cultivation patterns and microbial dynamics.
- **Areas for Improvement**:
  - A discussion of the limitations of the study and how future research can address them would add significant value. For example, the study is limited to the loess plateau—future research could explore whether the findings are consistent across other ecosystems.
  - The authors should recommend specific avenues for future research, such as examining the long-term effects of mixed planting systems on soil health or exploring the roles of other microbial groups (e.g., archaea, viruses) in ecological restoration.
  - The conclusion could also suggest more mechanistic studies that explore how specific plant-microbe interactions contribute to soil stability and nutrient cycling in mixed planting systems.

**Final Thoughts:**

- **Strengths**:
  - The concluding sentence appropriately emphasizes the broader ecological potential of the study’s findings, particularly regarding the stability of mixed forest systems.
- **Areas for Improvement**:
  - The final thoughts could be more assertive in stating the study’s contribution to the field of microbial ecology and ecological restoration. Instead of simply stating the potential of mixed forest systems, the conclusion could end on a more impactful note by suggesting that the findings should inform future restoration policies and land-use strategies.

### Recommendations for Improvement:

1. **Broaden the Implications**: The conclusion should link the study’s findings to broader ecological and practical applications, such as reforestation efforts or sustainable land management in degraded ecosystems beyond the study area.
2. **Restate the Ecological Significance**: Highlight the importance of the microbial functions identified in the study, such as carbohydrate metabolism, and how these functions contribute to long-term soil and ecosystem health.
3. **Address Limitations and Future Research**: Briefly acknowledge the limitations of the current study (e.g., geographic scope, short-term analysis) and provide specific recommendations for future research directions.
4. **Strengthen the Final Statement**: End the conclusion with a strong, assertive statement about the impact of the findings on future research, policy, or management strategies.

**References review**

The references section is comprehensive, although there are a few invalid citations (lines 407 [6-8], 442 [21, 22], and 481 [33-35]). The authors should correct these citations to ensure clarity and accuracy. These citations appear as placeholders and need to be corrected with the appropriate references or removed if unnecessary.

**Recommendation:**

I recommend **major revisions** to address clarity, enhance ecological relevance, and expand on the practical applications of the research. With these improvements, the manuscript has strong potential for publication.
