# Supplementary material for: Microbial community succession mediated by planting patterns in the Loess Plateau, China: Implications for ecological restoration
Source: PLoS One. 2025 May 27;20(5):e0324786. doi: 10.1371/journal.pone.0324786 (PMC12112373; doi:10.1371/journal.pone.0324786)
Supplement: Attachment — Submitted filename: Responses to Editor.docx [file pone.0324786.s004.docx]

Dear Editor,

Thanks for your comments. We have revised our manuscript carefully according to the request.

1. We have added Figures 1-10 to the manuscript as requested.
2. The source of Figure 1 has been clearly described. Source: Resources by Shuttle Radar Topography Mission Digital Elevation Model -90 meters resolution elevation data. <http://www.gscloud.cn> (revised in manuscript).
3. The data availability statement has been revised in the manuscript and updated in the cover letter.
