# Supplementary material for: Microbial community succession mediated by planting patterns in the Loess Plateau, China: Implications for ecological restoration
Source: PLoS One. 2025 May 27;20(5):e0324786. doi: 10.1371/journal.pone.0324786 (PMC12112373; doi:10.1371/journal.pone.0324786)
Supplement: Attachment — Submitted filename: Plos One_Revision_Review_Microbial_123 (Autosaved).docx [file pone.0324786.s003.docx]

**Review Report on Author Revisions - PONE-D-24-15257**

**Title:** Microbial community succession mediated by planting patterns in the loess beam-shaped hilly region of China

**Reviewer**: Bright Fafali Dogbey

**Overview of the Revisions**

The authors have responded comprehensively to the review comments, significantly improving the manuscript in **clarity, methodology, discussion, and data presentation**. Below is an assessment of how the authors addressed the initial concerns, along with any remaining issues requiring attention.

**Comparison of Revisions with Original Review Comments**

**1. Title & Abstract**

- **Comment:** The abstract is now more structured and highlights the study’s significance well. However, the methodology section in the abstract could be improved by briefly mentioning the sample size and experimental setup to provide better context.
- **Suggested Revision:**
  **Current sentence:**
  *This study utilized metagenomic high-throughput sequencing technology to investigate the functional dynamics of microbial communities in soil samples from different planting areas (single planting area to a mixed planting area) in the ecological degradation areas of the Chinese Loess Plateau.*
  **Revised sentence:**
  *This study utilized metagenomic high-throughput sequencing technology to analyze microbial community dynamics in soil samples collected from eight different planting patterns in the ecologically degraded areas of the Chinese Loess Plateau.*

**1. Clarity and Conciseness**

**Partially Addressed**

- The abstract is still somewhat wordy.
- The phrase *"is a core ecological issue for solving the problem of ecological degradation"* is redundant and could be simplified to *"plays a key role in addressing ecological degradation."*

**Remaining Issue:**

- The first two sentences could be restructured for better readability.
- Suggested Revision:
  **Original:**
  *The succession of microbial community structure and function in ecologically fragile areas is a core ecological issue for solving the problem of ecological degradation.*
  **Improved:**
  *Microbial community succession plays a key role in restoring fragile ecosystems and mitigating ecological degradation.*

| Review Comment | Adherence Level | Remaining Issues |
| --- | --- | --- |
| Clarity and Conciseness | Partially Addressed | Still wordy; first two sentences need refinement |
| Methodology Description | Partially Addressed | Sample size and details on sequencing depth missing |
| Key Findings and Statistical Relevance | Addressed but Needs Improvement | No statistical significance reported |
| Novelty and Implications | Addressed but Could Be Stronger | Needs clearer link to real-world applications |

**Final Verdict:** The authors have implemented some changes but need further refinements in clarity, methodological details, and statistical reporting.

**Introduction**

**1. Clear Statement of Research Gap**

**Partially Addressed**

- The introduction now includes a section discussing the limitations of previous research (*"most current research on soil microbial ecosystems focuses on specific species, habitats, forest types, or soil depths..."*).
- The authors acknowledge that studies on microbial structural composition and diversity *"driven by vegetation differences within small-scale areas remain limited."*

**Remaining Issue:**

- The specific research gap regarding microbial community succession under different planting patterns is still vague.
- **Suggested Addition:**
  *"Although previous research has explored vegetation restoration on degraded soils, the specific mechanisms through which different planting patterns affect microbial community succession and soil function remain unclear."*

**2. Hypothesis or Research Questions**

**Addressed**

- The authors included a hypothesis: *"Therefore, this study hypothesizes that mixed planting systems will promote greater microbial diversity and more effective soil functional restoration than monoculture systems."*

**Suggested Refinement for Clarity:**
**Current sentence:**
*"Therefore, this study hypothesizes that mixed planting systems will promote greater microbial diversity and more effective soil functional restoration than monoculture systems."*
**Suggested Revision:**
*"We hypothesize that mixed planting systems will enhance microbial diversity, increase functional gene expression, and improve soil enzyme activity more effectively than monoculture systems."*

- This makes the hypothesis more specific and testable.

**3. Streamlining Information on Past Programs**

**Partially Addressed**

- The authors have kept a discussion on the **Grain-for-Green Program (GFGP)** and its impact.
- They discuss challenges like **arbitrary selection of tree species and lack of consideration for vegetation adaptability** (lines 32-35).

**Remaining Issue:**

- The discussion on **deforestation, overgrazing, and previous restoration programs** is still somewhat lengthy.
- More focus should be placed on how these **land-use changes influence microbial communities** rather than just their general environmental effects.

**Suggested Refinement:**
**Current section (lines 26-36):**

- *Instead of detailing the entire history of deforestation, briefly summarize and transition directly to its effects on microbial communities and soil function.*

**Revised Example:**
*"The Loess Plateau has undergone significant degradation due to human activities, necessitating large-scale restoration efforts such as the Grain-for-Green Program (GFGP). However, while afforestation efforts have improved vegetation cover, their effects on microbial succession and soil health remain unclear. The arbitrary selection of tree species without considering microbial interactions may contribute to unintended ecosystem shifts."*

- This revision keeps the essential information while making it **more relevant to the study’s microbial focus**.

**4. Improving Readability of the Paragraph on Microbial Functional Importance**

**Addressed but Needs Refinement**

- The authors included a strong discussion on the role of **microbial communities in soil nutrient cycling, organic matter decomposition, and plant-soil feedback interactions** (lines 37-44).

**Remaining Issue:**

- **Lines 40-43 are overly complex.**
- The section starting with *"Various microorganisms with different functional characteristics in the soil..."* is still dense and could be simplified.

**Suggested Revision:**
**Current sentence:**
*"Although the proportion of soil microorganisms in soil quality is less than five thousandths (w/w), they play a key role in soil physiochemistry and biochemistry."*
**Revised:**
*"Despite comprising less than 0.5% of soil mass, microorganisms drive critical biochemical processes essential for soil fertility and ecosystem stability."*

- This eliminates redundancy and improves readability.

**Final Adherence Verdict**

| Review Comment | Adherence Level | Remaining Issues |
| --- | --- | --- |
| Clear Research Gap | Partially Addressed | Needs explicit statement on how planting patterns influence microbial succession |
| Hypothesis or Research Questions | Addressed | Could be more specific in linking diversity to functional traits |
| Streamlining Background on Restoration Programs | Partially Addressed | Still too much emphasis on deforestation, should focus more on microbial communities |
| Readability of Microbial Functions Section | Addressed but Needs Refinement | Some complex sentences should be simplified |
| Grammar & Readability | Partially Addressed | Overuse of commas and long sentences remain an issue |

**Final Verdict:** The authors have made significant improvements, but further refinements in clarity, conciseness, and grammar are needed.

**Materials and Methods Section**

**1. Study Area and Soil Sampling**

**Partially Addressed**

- The authors have revised the description of the study area, providing clearer details about its location and climate conditions.
- The term **"Liangmao hilly land in the belly of the Loess Plateau"** has been updated to **"the central region of the Loess Plateau"**, addressing my concern about clarity.

**Remaining Issues:**

- The **rationale for choosing 0–0.20 m soil depth** is still somewhat implicit.
  - They mention that **surface soils contain the most microbial activity and root exudates**, which is a valid point.
  - However, a **brief comparison with deeper soil layers** would strengthen this justification.
    **Suggested Addition:**
    *"While deeper soil layers may also harbor microbial communities, the 0-0.20 m depth was chosen as it is most influenced by plant root activity and soil disturbance."*

**Temporal vs. Spatial Variation** Explanation has been improved (lines 101–105).

- The authors now provide a **rationale for why spatial variations are more pronounced than temporal changes** in microbial community structure.
- This revision makes the methodology clearer and addresses my concern.

**2. Sampling Design and Procedures**

**Addressed Well**

- The **sampling method (three 20 m × 20 m plots per vegetation type, three 1.0 m × 1.0 m subplots per plot)** is clearly stated.
- The use of a **metal soil core sampler (15 cm inner diameter)** has been explicitly mentioned.

**Remaining Issues:**

- **Clarification of plant selection criteria:**
  - The phrase **"typical plants were selected"** (line 111) is deleted but authors claim it has been addressed.
  - Suggested addition if necessary:
    *"Representative plant species were selected based on dominance, ecological importance, and their role in the restoration process."*
- **Replication Justification:**
  - The **rationale for using three replicate plots** is still missing.
  - Suggested explanation:
    *"Three replicates per vegetation type were used to ensure statistical robustness and account for site-level variability."*

**3. Soil Physicochemical and Enzymatic Properties**

**Addressed Well**

- The authors have provided **references for standard soil analysis procedures** (e.g., dichromate oxidation for SOC, Kjeldahl for TN, and the molybdenum blue method for phosphorus).
- The **description of enzymatic assays** (sucrase, cellulase, urease, phosphatase, catalase, peroxidase, and polyphenol oxidase) is well-structured.

**Remaining Issues:**

- **Why were these specific enzymes chosen?**
  - The **functional relevance of these enzyme activities** is still not explicitly explained.
  - Suggested Addition:
    *"These enzymes were selected as indicators of soil microbial activity and nutrient cycling, which are essential for assessing ecological restoration progress."*
- **Equipment Specification:**
  - The authors now mention that the spectrophotometer used was **Metash V5100 (Shanghai, China)**.
  - This was one of my recommendations, and they have addressed it well.

**4. DNA Extraction and Sequencing**

**Addressed Well**

- The authors **retain the PowerSoil® DNA Kit** and provide a brief explanation of why it was used (lines 145–150).
- The **qRT-PCR methodology is well described**, including primer concentrations and thermal cycling conditions.

**5. Statistical Analysis**

**Partially Addressed**

- The authors have **provided better justification for Welch’s t-test**, mentioning its robustness for comparing samples with unequal variances (lines 177–179).
- The **ANOVA and post-hoc Duncan test** explanation is slightly improved.

**Remaining Issues:**

- **More justification for MetagenomeSeq over alternative tools**
  - While they list KEGG, eggNOG, and Pfam as functional annotation databases, they do not explain **why** MetagenomeSeq was used.
  - Suggested Addition:
    *"MetagenomeSeq was chosen due to its effectiveness in addressing sparse microbiome datasets, reducing bias in differential abundance analysis."*

**Final Adherence Verdict**

| Review Comment | Adherence Level | Remaining Issues |
| --- | --- | --- |
| Study Area Clarity | Mostly Addressed | Depth selection justification could be improved |
| Sampling Methods | Addressed | Plant selection criteria and replication justification still unclear |
| Physicochemical Properties | Mostly Addressed | Explanation of enzyme selection needed |
| Statistical Justification | Partially Addressed | Justification for MetagenomeSeq still weak |
| Sequencing Quality Control | Well Addressed | Addressed by adding FastQC and Fastp details |

**Final Verdict:** The authors have incorporated most of the recommendations, but minor clarifications in plant selection criteria, enzyme choices, sequencing rationale, and statistical justifications are still needed.

Revised Results and Discussion

**1. Soil Physicochemical and Biological Enzymatic Activities**

**Partially Addressed**

- The revised section **includes more details on soil parameters** and provides **comparisons between single and multi-planting systems**.
- The authors acknowledge **soil stability and aggregate formation as drivers of microbial diversity**, which strengthens the ecological relevance.
- They mention that **phosphatase, sucrase, BG, and NAG levels were significantly lower in the restored planting area than in the abandoned area** (lines 214–215).

**Remaining Issues:**

- **Clarity in Data Presentation:**
  - The **discussion on enzyme activity data** is still somewhat unclear.
  - **Suggested Addition:**
    *"Higher phosphatase and sucrase activities indicate enhanced microbial nutrient cycling, which is essential for soil restoration and plant nutrient availability."*
- **NO_3_–N and NH_4_^+^–N Variability Explanation:**
  - The **explanation for fluctuations in nitrogen forms across planting types** is still minimal.
  - **Suggested Expansion:**
    *"The observed increase in NH_4_^+^ and TN under restorative planting suggests that plant-soil interactions enhance nitrogen retention, while fluctuating NO_3_–N levels may indicate differential microbial uptake or leaching patterns."*

**Final Verdict:** Good improvements, but enzyme activity data interpretation needs more depth.

**2. Soil Microbiological Composition Under Different Planting Patterns**

**Mostly Addressed**

- The **dominance of Acidobacteria** is now explicitly discussed in relation to nutrient cycling, organic matter decomposition, and pH regulation (lines 242–245).
- The **relative abundance of bacterial taxa is presented well**, with a clear breakdown of percentages.

**Remaining Issues:**

- **More Direct Connection to Functional Roles of Microbial Taxa:**
  - While Acidobacteria’s importance is noted, the role of **other key bacterial groups** (e.g., *Gemmatimonadetes, Verrucomicrobia*) is not sufficiently explained.
  - **Suggested Expansion:**
    *"Gemmatimonadetes are commonly associated with drought tolerance and may contribute to microbial resilience in degraded soils. Verrucomicrobia have been linked to carbohydrate metabolism and organic matter decomposition, which are crucial for soil restoration."*

**Final Verdict:** Improved discussion on Acidobacteria, but other microbial groups need more attention.

**3. Functional Changes of Microbial Communities**

**Partially Addressed**

- The revised version **better explains the significance of functional gene annotations** (lines 267–273).
- The KEGG, eggNOG, and CAZy pathway results are now **more explicitly tied to microbial metabolism**.

**Remaining Issues:**

- **Functional Significance of Carbohydrate and Amino Acid Metabolism Needs More Detail**
  - The authors note that **amino acid and carbohydrate metabolism were dominant pathways** but do not fully explain why this is important for soil restoration.
  - **Suggested Addition:**
    *"The dominance of carbohydrate metabolism genes suggests enhanced microbial degradation of plant residues, facilitating soil organic matter accumulation. Similarly, increased amino acid metabolism may indicate active nitrogen cycling, which is critical for restoring degraded soils."*
- **Link Functional Gene Abundance to Microbial Community Structure:**
  - The authors state that **functional gene diversity correlates with microbial abundance**, but this link is not well explained.
  - **Suggested Addition:**
    *"Higher microbial diversity in mixed planting systems aligns with increased functional gene richness, suggesting that plant diversity promotes functionally diverse microbial communities."*

**Final Verdict:** Functional pathways are better discussed, but their ecological significance could be further expanded.

**4. Diversity Changes of Microbial Communities**

**Partially Addressed**

- The **β-diversity analysis (PCoA, NMDS, UPGMA, PERMANOVA)** is well presented, with clear mentions of microbial clustering patterns (lines 320–327).

**Remaining Issues:**

- **Lack of Explanation for Why Mixed Planting Enhances Microbial Stability:**
  - The authors state that **mixed planting promotes biodiversity and microbial stability**, but the mechanism remains unclear.
  - **Suggested Expansion:**
    *"Mixed plant species create varied rhizosphere environments that promote microbial niche differentiation. This reduces competition and supports more diverse microbial interactions, leading to ecosystem stability."*
- **More Depth on Correlation Between Microbial Diversity and Soil Health:**
  - **Suggested Addition:**
    *"Higher microbial diversity is linked to improved soil resilience, as diverse communities provide redundancy in ecological functions, ensuring stability under environmental stress."*

**Final Verdict:** Good improvements, but microbial diversity-stability links need more explanation.

**5. Discussion Section**

**Partially Addressed**

- The discussion **effectively summarizes key findings and connects them to ecological processes** (lines 296–319).
- **Mixed planting effects on soil fertility recovery** are clearly articulated.

**Remaining Issues:**

- **Stronger Connections Between Functional Shifts and Microbial Interactions Needed:**
  - While the discussion **mentions soil enzyme activity and microbial changes**, these aspects are not explicitly connected.
  - **Suggested Addition:**
    *"The increased abundance of sucrase and phosphatase activity in mixed planting areas suggests enhanced microbial decomposition of organic matter and phosphorus mobilization, contributing to soil restoration."*
- **Expand on Broader Ecological Implications:**
  - The **implications for long-term soil health and plant-microbe interactions** need more emphasis.
  - **Suggested Addition:**
    *"Understanding microbial succession under different planting patterns can inform sustainable land management strategies, particularly in degraded ecosystems."*

**Final Verdict:** The discussion is well structured, but explicit connections between microbial functions and soil health are still needed.

**6. Study Limitations and Future Directions**

**Partially Addressed**

- The authors acknowledge **limitations such as temporal variability and site-specific conditions** (lines 343–378).

**Remaining Issues:**

- **Future Research Directions Need More Specificity**
  - **Suggested Addition:**
    *"Future studies should focus on long-term microbial succession patterns, plant-microbe interactions, and the role of microbial inoculants in accelerating soil recovery."*

**Final Verdict:** Acknowledgement of limitations is good, but future research recommendations could be more targeted.

**Final Adherence Verdict**

| Review Comment | Adherence Level | Remaining Issues | Review Comment |
| --- | --- | --- | --- |
| Clarity in Data Presentation | Partially Addressed | Enzyme activity interpretation still unclear | Clarity in Data Presentation |
| Statistical Significance Emphasis | Addressed | Statistical comparisons improved | Statistical Significance Emphasis |
| β-Diversity Explanation | Partially Addressed | Needs better explanation of microbial stability | β-Diversity Explanation |
| Functional Gene Analysis | Partially Addressed | Link to microbial functions needs more depth | Functional Gene Analysis |
| Microbial Taxa Explanation | Mostly Addressed | Some key groups still not discussed | Microbial Taxa Explanation |
| Discussion Structure & Clarity | Partially Addressed | Stronger connections between microbial function and soil health needed | Discussion Structure & Clarity |
| Future Research Directions | Partially Addressed | Could suggest more concrete next steps | Future Research Directions |

**Final Verdict: The authors have made strong revisions, but further clarifications in enzyme activity significance, microbial diversity effects, and broader ecological implications would enhance the manuscript.**

Revised Conclusion Section

**1. Summary of Findings**

**Mostly Addressed**

- The conclusion now **summarizes key findings well**, particularly the effects of **mixed versus single planting systems on microbial diversity and soil properties** (lines 446–463).
- The **increase in microbial community abundance (32.1%) in mixed forest areas compared to single-planting areas** is a strong addition, adding quantitative support.

**Remaining Issues:**

- The **explanation of why mixed forests support higher microbial diversity** is still somewhat vague.
- **Suggested Addition:**
  *"The increased microbial abundance in mixed forests may be attributed to a more diverse range of root exudates, which provide varied carbon sources that support a broader microbial community."*

**Final Verdict:** Good improvements, but microbial diversity enhancement mechanisms could be clearer.

**2. Linkages to Broader Ecological Implications**

**Partially Addressed**

- The authors **mention the role of microbial communities in ecosystem stability and restoration**, which aligns with my suggestion to **strengthen the broader ecological significance** (lines 458–461).

**Remaining Issues:**

- The **practical applications of these findings for ecological management** are not explicitly discussed.
- **Suggested Addition:**
  *"These findings emphasize the importance of integrating mixed-planting strategies into land restoration projects, particularly in degraded ecosystems, to enhance soil microbial diversity and resilience."*

**Final Verdict:** The ecological relevance is addressed, but the practical applications for land management could be made more explicit.

**3. Addressing Future Research Directions**

**Not Addressed**

- The **conclusion does not outline future research directions**, despite this being one of my main suggestions.
- **Suggested Addition:**
  *"Future research should explore the long-term stability of microbial communities under different planting systems and investigate the interactions between plant root exudates and microbial metabolic pathways."*

**Final Verdict:** Future research directions are missing and should be added.

**4. Acknowledgment of Study Limitations**

**Not Addressed**

- The **conclusion does not acknowledge any potential limitations** of the study, which would help provide a balanced perspective.
- **Suggested Addition:**
  *"While this study provides valuable insights, it is limited by its short-term analysis. Future work should assess seasonal and long-term microbial dynamics to fully understand the sustainability of mixed-planting systems."*

**Final Verdict:** Study limitations should be briefly acknowledged.

**Final Adherence Verdict**

| Review Comment | Adherence Level | Remaining Issues |
| --- | --- | --- |
| Summary of Findings | Mostly Addressed | Mechanisms for microbial diversity enhancement need more detail |
| Broader Ecological Implications | Partially Addressed | Practical applications for land restoration need more emphasis |
| Future Research Directions | Not Addressed | No discussion of future research questions or gaps |
| Study Limitations | Not Addressed | Needs acknowledgment of study constraints and potential improvements |
| Review Comment | Adherence Level | Remaining Issues |

**Final Verdict:** The conclusion summarizes findings well but needs improvements in future research directions, study limitations, and practical applications.

**Revised Conclusion (More Concise & Impactful)**

**Original:**
*The composition and changes of microbial communities are influenced by many endogenous and exogenous factors. These factors may include temperature, humidity, oxygen concentration, nutritional status, land use patterns, environmental pollution, etc. Microbial communities change rapidly, and they can adapt to changes in the environment and respond accordingly to these changes. The microbial community is of great significance to the normal operation of the ecosystem, so in daily life and production, we should pay more attention to and protect the microbial community to avoid unnecessary interference.*

**Revised:**
*Microbial community composition and dynamics are shaped by various environmental factors, including temperature, moisture, oxygen levels, nutrient availability, and land use. These communities play a vital role in ecosystem stability, necessitating sustainable land management practices to preserve soil microbial diversity.*

**Original:**
*In this study, the effects of different restorative plantations on the soil microbial composition and structure were investigated. The topographic location and restorative planting patterns significantly affected the soil microbial quantities and functions. The abundance of soil C and N nutrients was the highest in the mixed forest soil, and the total number of microorganisms was the highest. Conversely, the soil microbial inventory in a single planting area was the lowest and existed as a single or few dominant species, indicating that the soil microbial community diversity in a single planting area was relatively low and could easily be lost.*

**Revised:**
*This study demonstrated that topography and planting patterns significantly influence soil microbial composition and function. Mixed forests had the highest soil carbon (C) and nitrogen (N) content, along with the greatest microbial abundance and diversity. In contrast, single-species planting systems showed lower microbial diversity, with fewer dominant species, making them more susceptible to biodiversity loss.*

**Original:**
*Due to the erosion process and loss of soil microbial diversity, the proportion of large aggregates in bare soil in single-variety planting areas was the lowest, and the carbon density of large aggregates and micro-aggregates was the smallest. Soil microbial community abundance in the mixed forest area was approximately 32.1% higher than that in the single forest area. This may be because the complex rhizosphere-mediated nutrient soil is more conducive to the reproduction of a variety of dominant microorganisms and the growth of multifunctional bacteria.*

**Revised:**
*Erosion and biodiversity loss were more pronounced in single-planting areas, where large soil aggregates had lower carbon density. Microbial abundance in mixed forests was 32.1% higher than in single-species plots, likely due to the more diverse rhizosphere environment supporting a broader range of microorganisms.*

**Original:**
*The detection of functional genes related to energy metabolism and species conversion showed that material and energy bases are important factors in maintaining the stability of degraded ecological environments. By improving the management and control of soil cultivation patterns, mixed forest areas have great potential for forming multiple dominant species, which is conducive to maintaining the stability of fragile ecological environments. Therefore, this study provides valuable data support for soil restoration in ecologically fragile areas in the future.*

**Revised:**
*Functional gene analysis revealed that energy metabolism and species interactions are key to stabilizing degraded ecosystems. Proper management of planting patterns, particularly through mixed forests, can enhance microbial diversity and ecological resilience. These findings provide valuable insights for soil restoration efforts in fragile ecosystems.*

**Key Improvements:**

**Reduced Redundancy** – Removed repetitive phrases like *"microbial communities change rapidly"* and *"we should pay more attention to protecting them."*
**Clearer Scientific Focus** – More emphasis on *why* mixed planting enhances soil health.
**More Direct and Concise** – Reduced word count while maintaining all essential findings.

**Final Verdict:**

**Revised Conclusion**

Microbial community composition and dynamics are shaped by various environmental factors, including temperature, moisture, oxygen levels, nutrient availability, and land use. These communities play a vital role in ecosystem stability, necessitating sustainable land management practices to preserve soil microbial diversity.

*This study demonstrated that topography and planting patterns significantly influence soil microbial composition and function. Mixed forests had the highest soil carbon (C) and nitrogen (N) content, along with the greatest microbial abundance and diversity. In contrast, single-species planting systems showed lower microbial diversity, with fewer dominant species, making them more susceptible to biodiversity loss.*

*Erosion and biodiversity loss were more pronounced in single-planting areas, where large soil aggregates had lower carbon density. Microbial abundance in mixed forests was 32.1% higher than in single-species plots, likely due to the more diverse rhizosphere environment supporting a broader range of microorganisms.*

*Functional gene analysis revealed that energy metabolism and species interactions are key to stabilizing degraded ecosystems. Proper management of planting patterns, particularly through mixed forests, can enhance microbial diversity and ecological resilience. These findings provide valuable insights for soil restoration efforts in fragile ecosystems.*

**Key Revisions & Justifications:**

1. **Conciseness & Readability**
   - Redundant phrases have been removed while maintaining all essential findings.
   - The revised version reduces wordiness while making the conclusion more direct and impactful.
2. **Stronger Scientific Focus**
   - The revised version explicitly states the role of **microbial communities in soil stability** rather than making general recommendations about their protection.
   - The relationship between **planting patterns and microbial function** is made clearer.
3. **Practical Implications for Soil Restoration**
   - The revised version emphasizes the **role of mixed planting systems** in ecological restoration and biodiversity conservation.
   - It highlights the practical relevance of the findings, making the study more applicable to land management strategies.
4. **Improved Flow & Logical Progression**
   - The revision presents the findings in a more structured manner: **first summarizing microbial responses, then explaining soil physical properties, and finally discussing functional genes and their ecological implications.**

**Additional Recommendations for Enhancing the Conclusion:**

- **Consider Including Future Research Directions:**
  - The conclusion could briefly mention **potential areas for further research**, such as long-term microbial succession monitoring or plant-microbe interactions under different planting systems.
  - Suggested addition: *"Future studies should explore the long-term stability of microbial communities under different planting systems and investigate the interactions between plant root exudates and microbial metabolic pathways."*
- **Acknowledgment of Study Limitations:**
  - A short acknowledgment of potential limitations (e.g., study duration, soil variability) would provide a balanced perspective.
  - Suggested addition: *"While this study provides valuable insights, it is limited by its short-term analysis. Future work should assess seasonal and long-term microbial dynamics to fully understand the sustainability of mixed-planting systems."*
