# Supplementary material for: Microbial community succession mediated by planting patterns in the Loess Plateau, China: Implications for ecological restoration
Source: PLoS One. 2025 May 27;20(5):e0324786. doi: 10.1371/journal.pone.0324786 (PMC12112373; doi:10.1371/journal.pone.0324786)
Supplement: Attachment — Submitted filename: Responses to Reviewers.docx [file pone.0324786.s006.docx]

**Dear Dr Massimiliano Cardinale**

Thank you and the two reviewers for the very constructive comments. We have revised our manuscript carefully following the comments and provide specific responses to each of the reviewers’ comments in blue. To make it easier for you to see where changes have been made in the manuscript, all major changes are in red.

**Overview of the Revisions**

The authors have responded comprehensively to the review comments, significantly improving the manuscript in clarity, methodology, discussion, and data presentation. Below is an assessment of how the authors addressed the initial concerns, along with any remaining issues requiring attention.

Response: Thank you for such positive comments and your valuable suggestions. We have carefully revised the manuscript and believe that it is now much improved.

**Title & Abstract**

1.The methodology section in the abstract could be improved by briefly mentioning the sample size and experimental setup to provide better context.

Response: We have made the suggested changes (revised manuscript). Revised sentence: *This study utilized metagenomic high-throughput sequencing technology to analyze microbial community dynamics in soil samples collected from eight different planting patterns in the ecologically degraded areas of the Chinese Loess Plateau.*

2.The abstract is still somewhat wordy. The first two sentences could be restructured for better readability.

Response: We have made the suggested changes (revised manuscript). Revised sentence: *Microbial community succession plays a key role in restoring fragile ecosystems and mitigating ecological degradation.*

**Introduction**

1. The specific research gap regarding microbial community succession under different planting patterns is still vague. Suggested Addition: *“**Although previous research has explored vegetation restoration on degraded soils, the specific mechanisms through which different planting patterns affect microbial community succession and soil function remain unclear."*

Response: We have made the suggested changes (revised manuscript).

2. Hypothesis or Research Questions. Suggested Refinement for Clarity. Suggested Refinement for Clarity: *"Therefore, this study hypothesizes that mixed planting systems will promote greater microbial diversity and more effective soil functional restoration than monoculture systems."*

Response: We have made the suggested changes (revised manuscript). Revised sentence: *We hypothesize that mixed planting systems will enhance microbial diversity, increase functional gene expression, and improve soil enzyme activity more effectively than monoculture systems.*

3. Streamlining Information on Past Programs. The discussion on deforestation, overgrazing, and previous restoration programs is still somewhat lengthy. More focus should be placed on how these land-use changes influence microbial communities rather than just their general environmental effects.

Response: We have made the suggested changes (revised manuscript). Revised sentence: *The Loess Plateau has undergone significant degradation due to human activities, necessitating large-scale restoration efforts such as the Grain-for-Green Program (GFGP). However, while afforestation efforts have improved vegetation cover, their effects on microbial succession and soil health remain unclear. The arbitrary selection of tree species without considering microbial interactions may contribute to unintended ecosystem shifts.*

4. Improving Readability of the Paragraph on Microbial Functional Importance.

Response: We have made the suggested changes (revised manuscript). Revised sentence: *Despite comprising less than 0.5% of soil mass, microorganisms drive critical biochemical processes essential for soil fertility and ecosystem stability.*

**Materials and Methods Section**

1.The rationale for choosing 0–0.20 m soil depth is still somewhat implicit. However, a brief comparison with deeper soil layers would strengthen this justification.

Response: We have made the suggested changes (revised manuscript). Revised sentence: *While deeper soil layers may also harbor microbial communities, the 0-0.20 m depth was chosen as it is most influenced by plant root activity and soil disturbance.*

2. Clarification of plant selection criteria: The phrase "typical plants were selected" (line 111) is deleted but authors claim it has been addressed. Suggested addition if necessary.

Response: We have made the suggested changes (revised manuscript). Revised sentence: *Representative plant species were selected based on dominance, ecological importance, and their role in the restoration process.*

3. The rationale for using three replicate plots is still missing. Suggested explanation: *"Three replicates per vegetation type were used to ensure statistical robustness and account for site-level variability."*

Response: We have made the suggested changes (revised manuscript).

4.Why were these specific enzymes chosen? Suggested Addition: *"These enzymes were selected as indicators of soil microbial activity and nutrient cycling, which are essential for assessing ecological restoration progress."*

Response: We have made the suggested changes (revised manuscript).

5.While they list KEGG, eggNOG, and Pfam as functional annotation databases, they do not explain why MetagenomeSeq was used. Suggested Addition: *"MetagenomeSeq was chosen due to its effectiveness in addressing sparse microbiome datasets, reducing bias in differential abundance analysis."*

Response: We have made the suggested changes (revised manuscript).

**Revised Results and Discussion**

1. The discussion on enzyme activity data is still somewhat unclear. Suggested Addition: *"Higher phosphatase and sucrase activities indicate enhanced microbial nutrient cycling, which is essential for soil restoration and plant nutrient availability."*

Response: We have made the suggested changes (revised manuscript).

2.NO_3_^-^–N and NH_4_^+^–N Variability Explanation.

Response: We have made the suggested changes (revised manuscript). Revised sentence: *The observed increase in NH_4_^+^ and TN under restorative planting suggests that plant-soil interactions enhance nitrogen retention, while fluctuating NO_3_–N levels may indicate differential microbial uptake or leaching patterns.*

3. While Acidobacteria’s importance is noted, the role of other key bacterial groups (e.g., *Gemmatimonadetes, Verrucomicrobia*) is not sufficiently explained.Suggested Expansion:*"Gemmatimonadetes are commonly associated with drought tolerance and may contribute to microbial resilience in degraded soils. Verrucomicrobia have been linked to carbohydrate metabolism and organic matter decomposition, which are crucial for soil restoration."*

Response: We have made the suggested changes (revised manuscript). Revised sentence: *Beyond Acidobacteria, Gemmatimonadetes and Verrucomicrobia also play crucial roles in soil ecosystems. Gemmatimonadetes are commonly associated with drought tolerance and may contribute to microbial resilience in degraded soils. Their ability to withstand low-moisture conditions suggests that they may play a role in maintaining microbial activity and functionality under drought stress. Verrucomicrobia, on the other hand, have been linked to carbohydrate metabolism and organic matter decomposition, which are crucial for soil restoration. Their enzymatic capabilities enable them to degrade complex organic compounds, contributing to nutrient recycling and improving soil fertility. The presence of these bacterial groups highlights the complex interactions within microbial communities and their collective influence on soil restoration and stability.*

4. The authors note that amino acid and carbohydrate metabolism were dominant pathways but do not fully explain why this is important for soil restoration.Suggested Addition: *"The dominance of carbohydrate metabolism genes suggests enhanced microbial degradation of plant residues, facilitating soil organic matter accumulation. Similarly, increased amino acid metabolism may indicate active nitrogen cycling, which is critical for restoring degraded soils."*

Response: We have made the suggested changes (revised manuscript).

5. Link Functional Gene Abundance to Microbial Community Structure: The authors state that functional gene diversity correlates with microbial abundance, but this link is not well explained. Suggested Addition: *"**Higher microbial diversity in mixed planting systems aligns with increased functional gene richness, suggesting that plant diversity promotes functionally diverse microbial communities."*

Response: We have made the suggested changes (revised manuscript).

6. Lack of Explanation for Why Mixed Planting Enhances Microbial Stability:

The authors state that mixed planting promotes biodiversity and microbial stability, but the mechanism remains unclear. Suggested Expansion: *"Mixed plant species create varied rhizosphere environments that promote microbial niche differentiation. This reduces competition and supports more diverse microbial interactions, leading to ecosystem stability."*

Response: We have made the suggested changes (revised manuscript). Revised sentence: *Mixed plant species create varied rhizosphere environments that promote microbial niche differentiation. This reduces competition and supports more diverse microbial interactions, ultimately leading to ecosystem stability. Different plant species exude different root exudates, including organic acids, sugars, amino acids, and secondary metabolites, which selectively enrich specific microbial populations. This process enhances functional complementarity among microbial taxa, reducing competitive exclusion and allowing a greater variety of microbes to coexist. Moreover, a diverse plant community increases soil structural complexity, providing microhabitats that further contribute to microbial diversity and resilience.*

7. More Depth on Correlation Between Microbial Diversity and Soil Health:

Suggested Addition: *"Higher microbial diversity is linked to improved soil resilience, as diverse communities provide redundancy in ecological functions, ensuring stability under environmental stress."*

Response: We have made the suggested changes (revised manuscript). Revised sentence: *Higher microbial diversity is linked to improved soil resilience, as diverse communities provide redundancy in ecological functions, ensuring stability under environmental stress. This redundancy allows the soil microbiome to maintain critical functions such as organic matter decomposition, nutrient cycling, and disease suppression even under adverse conditions. For instance, in degraded soils, a diverse microbial community can mitigate nutrient limitations by facilitating nutrient mobilization and enhancing plant-microbe interactions. Additionally, microbial diversity can improve soil aggregation, which enhances water retention and reduces soil erosion, further promoting ecosystem stability.*

8. Stronger Connections Between Functional Shifts and Microbial Interactions Needed: While the discussion mentions soil enzyme activity and microbial changes, these aspects are not explicitly connected. Suggested Addition: *"The increased abundance of sucrase and phosphatase activity in mixed planting areas suggests enhanced microbial decomposition of organic matter and phosphorus mobilization, contributing to soil restoration."*

Response: We have made the suggested changes (revised manuscript).

9. Expand on Broader Ecological Implications: The implications for long-term soil health and plant-microbe interactions need more emphasis. Suggested Addition: *"Understanding microbial succession under different planting patterns can inform sustainable land management strategies, particularly in degraded ecosystems."*

Response: We have made the suggested changes (revised manuscript). Revised sentence: *Understanding microbial succession under different planting patterns can inform sustainable land management strategies, particularly in degraded ecosystems. By studying how microbial communities respond to various cropping systems, it is possible to design agricultural and reforestation practices that enhance soil biodiversity and resilience. Long-term shifts in microbial composition influence nutrient cycling, organic matter accumulation, and the suppression of soilborne pathogens, all of which are essential for ecosystem restoration and productivity. Therefore, integrating microbial community dynamics into land management plans can provide a foundation for more sustainable and adaptive agricultural systems.*

**Revised Conclusion Section**

1. The author needs to revise the conclusion and outline future research directions as well as the limitations of the study.

Response: We have made the suggested changes (revised manuscript). Revised sentence: *Microbial community composition and dynamics are shaped by various environmental factors, including temperature, moisture, oxygen levels, nutrient availability, and land use. These communities play a vital role in ecosystem stability, necessitating sustainable land management practices to preserve soil microbial diversity. This study demonstrated that topography and planting patterns significantly influence soil microbial composition and function. Mixed forests had the highest soil carbon (C) and nitrogen (N) content, along with the greatest microbial abundance and diversity. In contrast, single-species planting systems showed lower microbial diversity, with fewer dominant species, making them more susceptible to biodiversity loss. The increased microbial abundance in mixed forests may be attributed to a more diverse range of root exudates, which provide varied carbon sources that support a broader microbial community. Erosion and biodiversity loss were more pronounced in single-planting areas, where large soil aggregates had lower carbon density. Microbial abundance in mixed forests was 32.1% higher than in single-species plots, likely due to the more diverse rhizosphere environment supporting a broader range of microorganisms. Functional gene analysis revealed that energy metabolism and species interactions are key to stabilizing degraded ecosystems. Proper management of planting patterns, particularly through mixed forests, can enhance microbial diversity and ecological resilience. These findings provide valuable insights for soil restoration efforts in fragile ecosystems. While this study provides valuable data support for future soil remediation in ecologically fragile areas, it is limited by its short-term analysis. Future work should explore the long-term stability of microbial communities under different planting systems and investigate the interactions between plant root exudates and microbial metabolic pathways, while assessing seasonal and long-term microbial dynamics to fully understand the sustainability of mixed-planting systems.*
