# Supplementary material for: Microbial community succession mediated by planting patterns in the Loess Plateau, China: Implications for ecological restoration
Source: PLoS One. 2025 May 27;20(5):e0324786. doi: 10.1371/journal.pone.0324786 (PMC12112373; doi:10.1371/journal.pone.0324786)
Supplement: Attachment — Submitted filename: Microbial Community Succession Revision Review Report.docx [file pone.0324786.s005.docx]

**Review Report on Revised Manuscript**

Title: Microbial Community Succession Mediated by Planting Patterns in the Loess Plateau, China: Implications for Ecological Restoration

Reviewer: Bright Fafali Dogbey

Manuscript Number: PONE-D-24-15257R2

General Comments: The authors have effectively addressed the majority of the concerns raised during the previous review, significantly enhancing the manuscript's clarity, methodological robustness, and scientific impact. Below is a detailed evaluation of their adherence to the previous comments, highlighting strengths and identifying a few remaining areas requiring minor adjustments.

Detailed Assessment:

1. Abstract:

- Improved substantially with the inclusion of clearer methodological details, explicitly mentioning sample size and the experimental setup.
- The abstract has also been refined for clarity and conciseness.

2. Introduction:

- The research gap is now explicitly defined, clearly indicating the limited understanding of how various planting patterns influence microbial community succession and soil functions.
- The hypothesis has been reformulated for clarity and specificity, effectively linking microbial diversity, functional gene expression, and soil enzyme activity.
- Background information on historical deforestation and restoration has been succinctly streamlined, focusing more clearly on microbial implications.

3. Methodology:

- The rationale for choosing the 0–0.20 m soil depth has been clearly justified.
- Plant selection criteria (dominance, ecological importance, and restoration roles) and the rationale behind plot replication have been comprehensively addressed.
- Justification for enzyme selection (as indicators of microbial activity and nutrient cycling) is explicitly provided.
- The choice of statistical methods (MetagenomeSeq) has been well justified and appropriately described.

4. Results and Discussion:

- Interpretations of enzyme activity data have improved significantly, explicitly linking activities to ecological restoration processes.
- The discussion on microbial diversity and its stability in mixed planting systems is substantially enhanced, clarifying the ecological mechanisms and implications.
- Functional gene analysis and its significance for soil restoration have been articulated more robustly.

5. Conclusion:

- The authors effectively summarized the key findings and clearly emphasized the broader ecological relevance of the study.
- The conclusion now explicitly addresses the ecological implications.

Final Recommendations: The authors have addressed the primary issues significantly, improving the manuscript's scientific quality and readability. It is recommended to accept the manuscript.

The manuscript is well-suited for publication in PLOS ONE.
